# Supplementary material for: Are carbon emissions trading and green financial instruments synergistic? -Comprehensive quantitative research based on content analysis
Source: PLoS One. 2024 Mar 7;19(3):e0298601. doi: 10.1371/journal.pone.0298601 (PMC10919601; doi:10.1371/journal.pone.0298601)
Supplement: S1 Data — (ZIP) [file pone.0298601.s001.zip › Data/地级市Z_F工作报告.zip!/╕╩╦α/╞╜┴╣╩╨/└┤╫╘╣1⁄2╓┌║┼í╛┬φ┐╦╩2╛▌═°í┐_2018.docx]

各位代表：

　　现在，我代表市人民政府向大会作工作报告，请予审议，并请政协委员和列席人员提出意见。

　　2017年工作回顾

　　今年以来，在市委的坚强领导下，在市人大、市政协的监督支持下，我们团结依靠全市人民，全面贯彻党的十八大、十八届三中、四中、五中、六中全会和十九大精神，深入学习贯彻习近平新时代中国特色社会主义思想和习近平总书记视察甘肃时的重要讲话及“八个着力”重要指示精神，统筹推进“五位一体”总体布局和协调推进“四个全面”战略布局，认真落实省第十三次党代会和市委四届四次全体会议精神，突出重点，真抓实干，克难奋进，基本实现了年度目标任务。

　　--经济保持平稳健康发展。认真落实“三重”“三一”工作方案，大力推行“五个一”产业发展模式，促使主要经济指标稳中有增、回升向好。预计到年底，全市地区生产总值完成392亿元，同比增长5%；一、二、三产增加值分别完成108亿元、96亿元和188亿元，增长5%、4%和5.5%；公共财政预算收入26.8亿元，同口径增长6%；社会消费品零售总额212亿元，增长9%。

　　--重大项目建设成效明显。以交通项目为带动，统筹推进水利、电力等基础设施建设，累计投资78.8亿元，平天、彭大高速和国道312线改造快速推进，平华一级、彬县至华亭等6条省道全线开工，建成农村公路199条1055公里；建设水利工程60处，泾川朱家涧、崇信关河等4座抗旱水库加快建设；±1100千伏特高压直流输电工程、750千伏变电站扩建等项目进展顺利。

　　--生态环境质量有效提升。扎实开展大气、水污染防治行动，投资20.7亿元，建成了一批环保基础设施，平凉城区环境空气优良天数达到323天，泾河、汭河、达溪河水质综合评价达到国家Ⅲ类水质标准，单位生产总值能耗、主要污染物排放控制在省政府下达目标值以内。大力推进国家森林城市创建，全市森林覆盖率提高到33.4%。我市被命名为国家生态文明建设示范市。

　　--开放开发步伐不断加快。积极“走出去”，学习先进经验，调整优化特色农业、旅游、工业化、城镇化发展思路和规划，引进签约了华崇灵高速、华亭热电联产集中供热、任丘节能炉具等项目，全市实施招商引资项目569项，落实到位资金389亿元。组团参加兰洽会、文博会、津洽会等重大节会，加强与天津市对口帮扶协作，成功举办了“金果红牛旅游”走进京津沪系列活动，签订农产品销售协议13亿元，平凉知名度进一步提高。

　　--人民获得感日益增强。脱贫攻坚取得阶段性成效，预计全市减少贫困人口8.7万人，贫困发生率下降到8.73%，全国打赢教育脱贫攻坚战现场会推广了静宁经验。新增城镇就业3.7万人。社会保障水平不断提高，全年发放各类保障金31.5亿元，受益498.1万人次。39万户群众看上了高清电视，21.6万农户接入高速光纤网络。预计全市城乡居民人均可支配收入分别增长8.2%、8.5%，达到25368元和7604元，居民消费价格涨幅控制在3%以内。

　　一年来，我们主要做了以下工作：

　　（一）因村因户施策，脱贫攻坚纵深推进。紧扣“六个精准”要求，聚焦深度贫困乡村，制定实施打赢脱贫攻坚战25条政策措施，实行市级领导联县包乡抓村制度，市级财政列支3000万元支持牛、果、菜产业发展，牛饲养量、出栏量分别达到123.5万头和47.3万头，新植果园22.8万亩、果品产量197万吨，新增设施蔬菜2万亩、蔬菜产量171万吨，推广旱作农业技术232.3万亩、粮食产量108万吨；推行“四个一”培训机制，培训输转劳务48万人，创收88亿元。整合项目资金38.2亿元，发放精准扶贫专项贷款8830万元，易地扶贫搬迁2845户1.1万人，改造农村危房7834户，建设撤并建制村通畅工程610公里，建成贫困村综合文化中心70个、日间照料中心74个、电商服务点120个，标准化村卫生室、有需求的行政村幼儿园实现全覆盖。农村低保与脱贫标准“两线合一”，精准认定农村低保对象6.7万户20.98万人，实现了应保尽保。

　　（二）强力推进重大项目建设，全力稳增长。建立重大项目“三个一”包抓责任制，联审联批，集中开工，推进重大项目建设，实施500万元以上项目1499个，其中，新集水库、平凉南铁路综合性货场、义乌商贸城等10亿元以上项目27个，平凉养生商贸城、鑫盛建材、庄浪中医院搬迁、泾川商品交易和农产品批发市场等过亿元项目226个。市县两级财政安排5000万元用于项目前期，2086个项目进入国家重大项目库，43个项目进入国省PPP项目库，全市项目库动态保持在5000亿元以上，邵寨煤矿等重大项目前期取得实质性进展；争取国省投资项目133项8.4亿元，融资127亿元。预计全市固定资产投资完成429亿元。

　　（三）坚持“四化”统筹，着力推进协调发展。把加快新型工业化、新型城镇化、农业现代化、信息化进程作为有机整体，统筹谋划、协调推进。围绕促进工业经济增长，全面落实“三去一降一补”，关闭煤炭企业1户、淘汰煤炭产能60万吨，“一企一策”推动33户停产半停产企业复产，华煤20万吨聚丙烯、赤城煤矿、天纤棉业20万锭棉纱项目加快建设，智能立体停车库生产基地、宝马纸业5万吨高速木浆纸、庆华陶瓷二期建成投产，庄浪、泾川等3个光伏项目并网发电，预计规上工业增加值完成47亿元，增长6.5%。深化农业供给侧结构性改革，加快发展现代农业，新建改扩建龙头企业25户，新登记注册农民合作社919个，新增家庭农场124个；海升超越农业12万平方米智能温室建成投产，泾汭河川区国家级蔬菜产业园等一批农业园区加快推进，全市农业科技贡献率达到56%；天源牧业等生态观光、游园采摘、农耕体验新业态快速兴起，农村一二三产融合步伐加快。着眼城乡一体化发展，集中开展棚户区改造“百日攻坚”行动，全市征收历年房屋尾欠2339户，开工改造棚户区8801户，一些长期影响项目建设的遗留问题得到有效解决。中心城区投资65亿元，实施生态宜居、公共服务、城市安全等建设项目60项，建成过街通道（天桥）3处；六县县城和重点小城镇实施基础设施建设项目206个，开建了一批医院、学校、体育场（馆）等公共服务设施，集聚能力不断增强，全市常住人口城镇化率达到40%。建成省级美丽乡村20个、市县级美丽乡村75个、环境整洁村200个。加快推进信息化进程，全光网城市全面建成，行政村、自然村光纤通达率分别为99.6%和70%；建成市政务云计算中心，电子政务全面推进；地理信息系统建设进展顺利，中心城区三维数字模型、城乡规划管理信息系统及三维地下管网系统建成启用，“雪亮工程”全国试点启动实施，信息技术应用更加广泛。

　　（四）多措并举，环境污染防治成效显著。坚决整改环保督察反馈问题，加快补齐环保基础设施短板。加强大气污染治理，投资7.9亿元，实施平凉热电联产集中供热二期和华亭县城区集中供热工程，关停治理城市建成区燃煤锅炉249台1877.6蒸吨，完成平凉电厂4号、6号机组超低排放改造，清理取缔散煤经销点294户，建成城市建成区优质煤专营市场和二级配送网点，淘汰黄标车3280辆；全面推行河长制，投资10亿元，加强集中式饮用水源地环境保护，实施葫芦河流域水环境综合治理项目，依法开展入河排污口排查整治，规范河道采砂，平凉中心城区、庄浪县生活污水处理厂扩容提标改造加快推进，华亭工业园区工业污水处理厂基本建成，平凉、静宁工业园区工业污水处理厂建成主体，全市新建污水收集管网85.8公里；扎实开展农用地土壤污染详查，投资6500万元，综合推进畜禽粪便、秸秆和废旧地膜资源化利用，搬迁禁养区内畜禽养殖企业44户；投资2.1亿元，启动实施全域无垃圾治理，推进农村环境连片整治，中心城区、华亭县城生活垃圾填埋场二期加快建设，5个重点乡镇生活垃圾填埋场建成投用，省级卫生城市创建通过验收。

　　（五）深化改革，发展活力不断增强。深入推进“放管服”改革，市级取消调整下放行政审批事项15项，市县两级非行政许可审批事项全部清零。全面推行“多证合一、一照一码”登记制度改革，全年新增非公经济市场主体1.6万户，581户个体户转型升级为企业。积极实施商标品牌战略，新增有效注册商标400个，地理标志商标达到9件。实施平凉工业园区体制机制改革，剥离社会事务职能，强化经济管理和投融资职能，功能定位更加明确。七县区农村土地承包经营权确权登记颁证工作通过省级验收，农村土地“三权分置”稳步推进，农村“三变”改革启动实施，累计流转土地99.8万亩。集体林权制度配套改革和水权水价、国有林场、供销社综合改革进展顺利。普惠金融服务体系进一步完善，兰州银行、银河证券、融兴村镇银行、甘肃省股权交易中心在平凉设立分支机构，华亭、庄浪、静宁3家农商银行成功改制，筹资6050万元分别搭建了农业信贷担保和小微企业“助保贷”平台，全市实现直接融资19.1亿元，金融业增加值占地区生产总值比重达到8.2%。

　　（六）突出普惠共享，民生保障水平持续提升。财政用于民生的支出占总支出的84%。省列10件实事全面完成，市列14件实事基本完成。全民参保登记工作全面推开，机关事业单位养老保险入轨运行，城镇职工异地就医直接结算全面实现。强化资金配套，提高保障标准，企业退休职工养老金增长7.3%，7.3万城乡居民和企业职工享受养老金14.2亿元；城镇居民医疗保险、新农合政府补助标准分别提高到620元和450元，为463.1万人次报销住院费11.2亿元；城乡低保标准分别提高8%和22.6%，为26万人发放保障金6.1亿元；全面落实“双创”政策，应届普通高校毕业生就业率达到90.1%，城镇登记失业率3.5%；推进保障性住房建设，完成老旧小区改（扩、翻）建31处、受益1.7万人。静宁县义务教育均衡发展通过国家评估认定，陕师大平凉实验中学建成招生，平凉职业技术学院完成整体搬迁。公立医院综合改革全面推开，分级诊疗模式加快建立，市级儿童专科病区和14个县级、11个乡镇医疗卫生机构建设项目进展顺利，医联体建设全面启动。全域旅游示范区创建扎实推进，打造了海寨沟、汭龙堡、锦绣凤凰等一批特色旅游景点，市博物馆建成主体，建成乡村舞台190个、乡镇（社区）体育健身中心8个、行政村体育惠民工程120个。严格落实安全生产责任制，切实强化食品药品安全监管，全面落实防灾减灾措施，深入推进社会治安综合治理，全市社会大局和谐稳定。民族宗教、统计调查、公共资源管理、档案、外事、侨务、地震、气象、人防、地方志、红十字、老龄、残疾人等事业取得新的成效，双拥共建、国防动员、民兵预备役等工作深入开展。

　　一年来，我们牢固树立 “四个意识”，坚定不移维护以习近平同志为核心的党中央权威和集中统一领导，认真贯彻执行中央、省市委重大决策部署。自觉接受人大法律监督、工作监督和政协民主监督，坚持重大事项向人大报告、向政协通报制度，充分听取各民主党派、工商联以及无党派人士意见建议，办理答复人大代表建议93件、政协委员提案117件。积极推进政府职能转变和管理创新，全面推进依法行政，不断健全重大行政决策公众参与、专家论证、风险评估、合法性审查和集体讨论决定制度，法治政府建设进程加快。扎实推进“两学一做”学习教育常态化制度化，大力开展党建统领“一强三创”行动，认真落实党风廉政建设主体责任，严格执行中央八项规定精神，强化审计监督和国家监察，严肃查处发生在群众身边的“四风”和腐败问题，持之以恒改进工作作风，政府自身建设不断加强。

　　各位代表，一年来，全市经济社会发展取得的成绩来之不易，这是省委、省政府和市委坚强领导的结果，是市人大、市政协监督支持的结果，更是全市人民共同奋斗的结果。在此，我代表市人民政府，向辛勤工作在各条战线的广大干部群众，向中央、省驻平单位和驻平部队，向各民主党派、工商联、无党派人士，向离退休老同志，向关心、支持平凉建设和发展的各界人士，致以崇高的敬意和衷心的感谢！

　　同时，我们也清醒地认识到，受宏观经济下行影响，项目投资增量不足，民间投资大幅下滑，地区生产总值、固定资产投资等指标没有达到预期，全市经济社会发展中还有不少困难和问题，发展不平衡不充分的矛盾十分突出，政府工作中还存在着一些差距和不足。一是经济总量小、质量不高。全市地区生产总值不足400亿元，人均不到2万元，低于全省平均水平，仅为全国的三分之一，县域经济发展滞后。农业产业发展层次低，产业链条短，加工转化能力弱，规模、品牌和市场竞争力不强；工业经济薄弱，煤电产业全链式开发和深度加工不够、附加值不高，战略性新兴产业成长发育缓慢，工业投入严重不足，结构单一；文化旅游产业市场化运作滞后，服务业规模层次较低。二是交通瓶颈制约突出，基础设施建设滞后。对外交通缺少快速通道，境内无机场、没高铁，路网密度低，交通基础设施建设欠账大；城市内部交通不便捷、不顺畅，公共服务设施不健全，排污排洪、垃圾处理、供暖供气等设施不配套，城镇综合服务功能不强；水利基础薄弱，资源性、工程性、水质性缺水问题并存。三是脱贫攻坚任务艰巨，民生领域还有不少短板。全市小康建设整体水平低，还有深度贫困村401个，16.99万人尚未脱贫，增收产业培育不足，“造血”功能不强，稳定脱贫基础不牢固。城乡优质教育资源不均衡，基层医疗服务能力较弱，食品药品、安全生产等方面还存在一些薄弱环节，部分低收入群众生活还比较困难，一些关系群众切身利益的问题还没有得到很好解决。四是城镇化水平低，辐射带动能力弱。对城镇长远发展战略研究不够，规划建设前瞻性不强，特色不够鲜明，功能布局不够合理；山水资源开发利用和景观打造不足，基础设施和公共服务设施配套不够完善，城市拥堵、停车难等问题突出，综合承载和服务能力不强；经营城市的思路不活、措施不多，市场运作、多元投入的渠道不畅。五是思想解放不够，干事创业活力不足。受传统惯性思维的影响，一些干部站位不高、理念不新、思想保守、安于现状，缺乏改革创新的魄力和能力，抓工作的视野不宽、办法不多，推动发展的意识不强；一些干部不敢为、不会为、不想为，懒政怠政，作风不实，落实不力。六是政府职能转变还不到位，发展环境营造不充分。“放管服”改革的协同性联动性不够，基层承接能力较弱；营商环境不优，开放程度低；一些政府部门单位主动服务的质量和效率不高，集成化办事的体制机制尚未形成。我们将正视发展中的困难、工作中的差距，采取坚决有力措施加以解决。

　　2018年工作总体要求和主要任务

　　2018年是贯彻党的十九大精神的开局之年，是决胜全面建成小康社会、实施“十三五”规划承上启下的关键一年。政府工作的总体要求是：全面贯彻党的十九大精神，以习近平新时代中国特色社会主义思想为指导，深入贯彻习近平总书记视察甘肃时重要讲话及“八个着力”重要指示精神，坚持稳中求进工作总基调，践行新发展理念，按照高质量发展要求，统筹推进“五位一体”总体布局和协调推进“四个全面”战略布局，坚持以供给侧结构性改革为主线，统筹推进稳增长、促改革、调结构、惠民生、防风险各项工作，认真落实市委四届四次全体会议部署要求，按照“党建统领、‘四化’统筹、交通先行、产业支撑、决战脱贫、决胜小康，建设绿色开放幸福美好新平凉”的发展思路，坚持问题导向，推动创新突破，提升运筹能力，弥补短板弱项，加快全面脱贫全面小康进程。

　　经济社会发展主要预期目标是：地区生产总值增长6.5%，规模以上工业增加值增长8%，固定资产投资增长9%，社会消费品零售总额增长8%，公共财政预算收入同口径增长7%，城乡居民人均可支配收入均增长8%，新增城镇就业3万人，城镇登记失业率控制在4%以内，居民消费价格涨幅3%左右，单位生产总值能耗和主要污染物排放完成省上控制目标。

　　围绕上述思路目标，重点做好九个方面的工作：

　　（一）强化产业支撑，打好脱贫攻坚战。聚焦精准扶贫，围绕实现“两不愁三保障”，坚持用工业化理念谋划农业，实施乡村振兴战略，全面落实25条政策措施，确保泾川、灵台、崇信、华亭四县脱贫摘帽，全市8.15万贫困人口脱贫退出。

　　集中发展以牛果劳为重点的增收产业。坚持把发展产业作为脱贫攻坚的首要任务，围绕创建国家级出口食品农产品质量安全示范区、泾汭河川区国家级蔬菜产业园区和全市现代农业产业园，按照“五个一”产业发展要求，对接落实全省1000亿元特色产业发展工程贷款和500亿元产业发展投资基金，用好3050万元农业信贷风险担保基金和3000万元牛果菜产业发展专项资金，大力推广“国有化农发公司+合作社+农户”庄浪模式，培育壮大农村新型经营主体，积极发展产业化联合体，加快推进全产业链开发，新建标准化养殖小区30个，牛饲养量124万头，新植果树经济林20万亩，蔬菜产量170万吨以上，因地制宜培育马铃薯、中药材等区域性特色产业，“三品一标”农产品达到54%，农产品加工转化率达到59%，新增农民合作社、家庭农场各100个，新建、改扩建龙头企业25户，加快构建现代农业产业体系、生产体系、经营体系，实现建档立卡贫困村增收产业、贫困户增收项目全覆盖，促使小农户与大市场有效对接。加强劳务协作，建好劳务基地，健全中介组织，扩大组织化输转，全年输转劳务47万人，创收90亿元。

　　加快补齐基础设施和公共服务短板。争取全省1000亿元农村基础设施和人居环境综合治理工程专项贷款，改造县乡公路150公里，建成自然村硬化路2600公里、村内主巷道670公里，实施县乡道安全生命防护工程470公里，改造农村危房7323户，易地扶贫搬迁4064户1.65万人。加快贫困村幼儿园、标准化村卫生室配套完善和综合公共文化服务中心建设。加大重特大疾病救助和农村低保、五保供养力度，兜牢社会保障底线。重视解决非贫困乡村基础设施、公共服务等问题，防止出现贫困“死角”。

　　深入推进农村改革。落实土地二轮承包延期三十年政策，加快实施“三权分置”改革。全面启动农村集体产权制度改革，泾川县要完成试点任务。积极推进自然资源统一确权登记，加快实施农村房地一体化确权颁证工作。紧扣摸清可变资本、选准发展产业、确定承接主体等关键环节，大力推进农村“三变”改革，有效活化资源资产，增加农民财产收益，壮大村级集体经济，上半年完成庄浪、崇信两县省市级试点任务，下半年全面推开。扩大政策性农业保险覆盖范围，减少农民因灾损失。推进集体林权制度配套改革，深化供销社综合改革。

　　着力激发内力用好外力。坚持扶贫与扶志、扶智相结合，发挥贫困户主体作用，创新扶贫方式，促进扶贫由“输血”向“造血”转变。全面落实教育扶贫政策，强化控辍保学措施，阻隔贫困代际传递。推行劳动力“四个一”培训机制，提高贫困群众发展生产、务工经商的基本技能，加快提升自我发展能力。加强农村“三个带头人队伍”建设，典型示范、能人带动，激发贫困群众脱贫的内生动力。强化驻村帮扶工作队作用，完善帮扶工作考评体系，深化定点扶贫、东西扶贫协作帮扶机制，鼓励各类社会组织开展到村到户精准扶贫，构建大扶贫格局。

　　（二）突出项目带动，增强发展后劲。充分发挥投资对稳增长、调结构、补短板的关键作用，落实重大项目建设“三个一”包抓责任制和清单式管理制度，坚定不移抓大项目、大抓项目，全年固定资产投资达到460亿元以上。

　　高标准谋划实施项目。按照“八个一批”项目谋划要求，深入对接国家政策导向和投资方向，建立项目前期经费增长机制，突出发展实体经济、壮大县域经济、特色产业开发、生态环境治理、保障改善民生等重点领域，精心谋划论证，做深做细前期，全市项目储备库动态保持在5000亿元以上，其中工业项目占到一半以上。年内实施500万元以上项目938项，其中，5000万元以上299项，亿元以上217项，10亿元以上23项；交通水利等基础设施项目149项143.6亿元，产业开发项目387项154.6亿元，城建及房地产项目222项160.9亿元，生态环保项目40项10.4亿元，社会事业项目141项46.8亿元，国省投资增长15%以上。

　　大力度开展招商引资。研究用好陕甘宁革命老区支持政策，完善招商引资优惠措施，以工业园区（集中区）为主阵地，突出延链招商、以商招商、点对点精准招商，坚持大中小齐抓、引资引智引技并重，着力提高招商引资项目落地率、资金到位率、投产达标率。建立重点招商项目全程服务、落地奖补制度，强化跟踪落实，开工建设27万台环保节能炉具制造、45万吨有机废弃物综合利用项目，力促光伏铸锭、3万吨铸造件生产线、城乡垃圾收集处理及发电等项目落地。鼓励支持县区、部门“走出去”、争项目、引投资。全年招商引资到位资金增长16%以上。

　　全方位保障项目落地。坚持用市场化的理念运筹项目，发挥财政资金和项目投资的撬动作用，推进政府和社会资本合作，实施一批PPP项目。市级财政列支500万元，组建专门工作班子，聘请专业团队策划规划，以PPP模式强力推进市体育运动公园建设。做大做强惠民、泓源、富民、文旅投等投融资平台，支持重点项目扩大股权和债券融资，鼓励民间资本进入医疗、养老、教育等民生领域，吸引更多金融资本、社会资本参与项目建设，有效破解资金难题。用足用活国家和省上支持深度贫困地区用地倾斜政策，着力解决影响项目建设的土地征用问题，加大闲置低效用地盘活力度，提高土地投资强度。优化审批流程，推行并联审批、集中审批、网上审批，打通项目前期“最后一公里”。

　　（三）坚持交通先行，加快破解发展瓶颈制约。聚焦基础设施短板，积极创造条件，争取国省支持，全力实施一批交通、环保、水利、电力项目，夯实发展基础。

　　聚力推进交通提升建设。组建专门班子，加快平凉机场、兰州至天水至平凉动车客运、定平庆高铁前期，协调推进宝中二线、西平二线前期。加快平天、彭大、华亭至泾源高速建设，开工建设静庄高速，华崇灵高速3月份开工，平华一级公路试验段基本建成，推进国道312线凤口至平凉段、彬县至华亭、崇信至方堤口等6条国省道建设，升级改造段家官庄至崆峒等3条普通省道，下决心建设内外联通的快速通道。完成3418公里自然村通硬化路工程前期并启动建设，为“三年任务两年完成”奠定基础。

　　坚决打好污染防治攻坚战。持续推进燃煤电厂超低排放改造，全部淘汰中心城区和县城建成区燃煤小锅炉，打赢蓝天保卫战。全面推行河长制，强化泾河、葫芦河等流域水环境综合整治，加强城市黑臭水体治理，加快县城污水处理厂扩容提标改造和城区污水管网配套，3个省级工业园区污水处理厂全面投入运行，开建4个工业集中区污水处理厂，建成乡镇垃圾填埋场28个、污水处理厂（站）26个。落实畜禽养殖禁养区禁养措施。强化分类指导，做好河道采砂、矿山采石规划，集中改造建设采砂厂，合理有序开采利用。持续推进全域无垃圾治理，继续开展城镇乡村、景区景点等十大环境卫生治理行动，着力改善城乡环境面貌。加大环境保护执法监管力度，坚决打击环保违法行为。深入推进“煤改气”“煤改电”等清洁化改造，大力开展节能技术改造和工程减排、结构减排、管理减排，推进节能、节水、节地、节材，促进资源全面节约和循环利用。

　　提升水利电力保障能力。推进引洮二期静宁城乡供水配套工程、新集水库等重点水利工程，加快白龙江引水工程和崆峒水库改扩建、泾川盘口、庄浪梁河等骨干水源工程前期；开建静宁成纪330千伏变电站，升级改造县区配电网，新建、改造10千伏及以下线路350公里。

　　（四）实施工业强市战略，加快新型工业化进程。紧盯工业发展“六大任务”，依托园区平台和大型企业，优化存量，扩大增量，加快构建以煤电化冶为主导，农产品深加工、新型建材、装备制造、战略性新兴产业竞相发展、协调共进的产业体系。

　　强化工业园区（集中区）平台载体作用。突出差异化、特色化、专业化，加快推进园区（集中区）体制机制改革，进一步明晰功能和产业定位，配套完善道路、供电、供水、环保、金融等服务设施，提升园区承载能力。加快平凉工业园区“瘦身提质”，实行市区合力共建、项目全市共引、“飞地经济”县区共推、开发建设成果共享，实施配售电改革和增量配电网试点，落实大用户直购电政策，全力争取100万吨电解铝及铝深加工项目落地建设，推动智能立体停车库生产基地尽快量产，建成义乌商贸城三期主体和平凉南铁路综合性货场一期工程，加快天然气支线管道及供气综合利用项目建设，打造全市产业综合型园区。加快建立华亭工业园区“飞地”税收分成机制，推动六县一区煤化工项目集中布点建设，力争20万吨聚丙烯尽早建成投产，争取开建二甲醚、甲醇扩能及烯烃转化、甲醇汽化渣场项目，打造全市新型煤化工产业园区。静宁工业园区围绕苹果产业开发，大力发展纸箱包装、农产品精深加工业，加大技术改造和产品创新，配套发展现代物流产业，建成在全国更有影响力的纸制品包装产业基地。其它4个工业集中区年底前全面完成改革工作，立足发展定位和资源优势，建成各具特色、支撑县域经济发展的增长极。全市工业园区（集中区）年内新入园亿元以上项目10个，千万元以上项目20个，完成投资36亿元。

　　依托大型企业集团扩大总量。按照“稳步扩煤、加快转电、突破化工”的思路，高标准编制煤电化冶产业发展规划，发挥华能、中电建、酒钢等企业集团的龙头带动作用，支持企业围绕煤电化冶延链补链、扩大投资、新上项目。建成赤城煤矿，推进五举煤矿减量置换，邵寨煤矿复工建设，加快唐家河煤矿、华亭电厂二期、陇东电力外送通道、40亿立方米煤制天然气项目前期工作，启动华煤煤制乙二醇、无纺布等4个项目前期，加快建设陇东国家级传统能源综合利用示范区。

　　支持非煤产业调优结构。建立5000万元市级工业发展专项资金，加快编制中国制造2025平凉发展规划，实施红峰蒸汽疏水阀系列产品智能化检测升级改造、虹光磁控管与高频无极灯军民融合项目，推动杭萧钢构、20万锭棉纱等30个项目建成投产，众一兴旺管材、贡禾食品二期等12个项目达产达标，着力提升装备制造、建材陶瓷、轻工纺织等传统产业水平。加快建设光伏、碳纤维、生物制药、煤机高端装备产业园，建设村级光伏扶贫电站265个，战略性新兴产业增加值增长10%以上。实施企业梯次培植计划，新增中小企业200户以上；落实入规企业户均10万元奖补政策，规下转规上企业10户。

　　（五）创新城市经营机制，加速新型城镇化建设。紧扣城市发展“四个转变”“六项原则”，明确目标方向、聚焦重点关键，着力构建以中心城市为龙头、县城为节点、重点小城镇为纽带、中心村为基点的城镇体系。

　　高标准编制城乡发展规划。深入研究城镇发展方向、空间结构、功能布局、产业支撑、风貌特色等重大问题，开展平凉城市总体规划评估，完成平凉市城市发展战略规划和中心城区总体城市设计编制，加快城市功能修补和生态修复、海绵城市、泾河水系景观等专项规划编制，全面推进市县“多规合一”城乡统筹总体规划编制，县城、重点小城镇控制性详细规划、重要基础设施专项规划实现全覆盖，持续做好乡村规划修编，形成系统完整的城乡规划体系，实现城镇开发和项目实施的全过程管控。

　　多途径经营运筹城镇。出台建设用地容积率管理办法，建立重点地段、区域、节点“土地出让+城市设计”制度，调节做优城市环境，凸显开发增值预期，吸引社会资本开发建设。严格实行增量供给与存量挖潜相结合的供地用地政策，加大土地置换和储备力度，健全房地产调控机制和未开发土地调规转向机制，拓宽土地升值空间。整合分散运营的城市资源，将热、水、气、商砼等有形资产和城市品牌、经营权、冠名权等无形资产打包进入融资平台，探索经营医疗、教育、养老等社会资源的路子，放开城市基础设施、公共服务设施建设市场，为加快城市发展聚集资金。

　　加快建设中心城市、县城、重点小城镇。发挥中心城市辐射带动作用，市级财政列支500万元开展泾河水系景观工程前期，依托泾渭河流域百万亩生态防护林基地项目，开工建设景观工程一期；放大“棚户区改造+”效应，实施棚户区改造2.7万户，其中老旧小区改（扩、翻）建2.4万户，开建新民北路、永兴路、永顺路、临泾路，建成新科路、天馨路，打通城市“断头路”，改善城市“微循环”；加快配套教育、卫生、文体服务设施，基本建成集中供热二期工程，开建平凉客运枢纽站、快速客运站，建设公共停车场（库）15处、新增停车位2000个，建成便民市场5处，整治城区人行道，修缮街巷道路，提升火车站、汽车站等重要节点形象。加快县城建设步伐，突出空间形态塑造、历史文化传承、山水特色展现，提升县城品位，尽快补齐供热供气、道路交通、污水垃圾处理等基础设施短板，规划实施一批公园广场、休闲步道和体育健身场所，培育各具特色、协同互补的产业支撑，推进产城融合。突出12个全国重点镇，整体出让基础设施建设、土地开发、城市管理等权益，加快培育特色小城镇。大力推进“厕所革命”，加强城区公厕和景区星级厕所建设管理，加快农村厕所“旱改水”步伐。实施“千合农庄计划”项目，建设美丽乡村100个、环境整洁村200个，打造一批集循环农业、创意农业、农事体验于一体的田园综合体。推进国家森林城市创建，持续抓好城乡重点区域绿化，全年植树造林50万亩。

　　加快推进农业转移人口市民化。实行城乡统一的户籍管理制度，结合易地搬迁和棚户区改造，引导农业人口通过打工、经商、创业向中心城市、县城、重点小城镇转移，就近就地市民化。打破政策壁垒，积极推进转移人口与城镇居民在上学、就医、就业等方面享受同等待遇，加快城镇常住人口基本公共服务全覆盖。加强农业转移人口教育培训，引导群众自觉遵守市民公约，提升转移人口城市生活能力，转变生产生活方式，更好地融入城镇发展。推进华亭撤县设市，全市撤乡改镇10个，常住人口城镇化率达到43%以上。

　　（六）打造全域旅游新品牌，做强区域首位产业。挖掘文化资源，讲好平凉故事，推进全域旅游，建设全景平凉。

　　加快旅游体制机制改革。成立市旅游发展委员会，强化产业规划、综合监管职能。按照所有权、管理权、经营权“三权分离、一步到位”原则，加快组建关山大景区管理机构，理顺崆峒山、大云寺·王母宫大景区管委会运行体制，完成崆峒山景区资源评价，引进实力雄厚、市场运作水平高的战略合作伙伴参与旅游资源开发和景区经营管理，促使旅游投资主体多元化，走出一条资源集成、开放开发的路子。

　　推进大景区建设。市级财政列支1600万元支持旅游产业发展，高标准编制平凉市全域旅游规划和关山大景区规划，建成泾川至田家沟景区连接公路，开建柳树沟至崆峒山、蔺家沟至莲花台2条景区连接公路，加快实施泾河南岸景观带、中华崆峒养生地养老中心、平凉原生态民俗文化体验区、崆峒生态旅游度假山庄等旅游基础设施和配套服务项目，开发朝那湫、五龙山-唐帽山等景区景点，扩大旅游景区体量，增强可进入性和驻足性，延长旅游产品服务周期，打造平凉旅游大龙头、大品牌。

　　培育旅游新业态。突出精品景区、养生产业园和乡村旅游示范点，加快创建一批新型旅游景区，抓建安口陶瓷、界石铺红色圣地等一批特色旅游小镇，实施乡村旅游“六小工程”，建设专业旅游村7个。推进崆峒区国家级全域旅游示范区、泾川县省级全域旅游示范县创建。精心谋划“塑造‘神奇崆峒·养生平凉’旅游整体形象”宣传推介活动，办好平凉崆峒养生文化旅游节、国际崆峒武术节等品牌节会赛事，扩大平凉旅游知名度。

　　促进文旅产业深度融合。围绕挖掘崆峒山道源文化、古成纪伏羲文化、西王母远古文化、皇甫谧医学文化品牌资源，抓建一批骨干文化企业，推出一批地域文化精品力作，加快中华崆峒养生地和陇东南国家中医药养生保健旅游创新区建设，推广针灸理疗、国医保健、健康养老等养生服务，开展养生文化旅游商品创意研发，发展具有平凉地域文化特色、创意理念先进、可批量生产的养生文化旅游商品，提升文化旅游产业竞争力。

　　（七）推进“智慧平凉”建设，提升信息化水平。加快大数据、物联网、云计算等新技术应用创新，打造一批面向政府、企业、民众的智慧应用系统，推进智能应用在各领域全面拓展。

　　完善信息基础设施。落实“宽带中国”战略，优化骨干网络架构，城乡宽带网络覆盖率达到100%。推进城乡网络增宽提速，全市互联网总出口宽带扩容至1400G，城市区域网速达到200兆，农村普及100兆，90%的自然村实现光网覆盖，50%以上农户和90%以上城镇家庭接入高速光纤网络。新建4G基站975个，4G网络实现城乡全覆盖。

　　建设大数据平台。全面梳理整合全市各类基础资源，建设覆盖人口、法人、地理、旅游、信用等资源的全市大数据公共服务平台，推进服务协同和资源共享。建设集运营管理、应急指挥、综合治理于一体的城市综合运营指挥中心，打造可视化平台，运用大数据对城市运行状态进行监测、分析预警和辅助决策。投资1.57亿元，实施“雪亮工程”。推进市政务云计算中心扩容升级，加快网上审批及电子监察系统应用， 提高市场监管、社会治理和公共服务的精准性、有效性。

　　实施“互联网+”行动。坚持信息化与工业、农业、服务业融合发展，构建“互联网+”现代农业、装备制造业、公共服务、现代物流、文化旅游、精准扶贫等新经济形态。加快推进电子商务进农村综合示范县建设，改建提升乡镇电商服务站和物流配送服务站20个，新建村级电商服务点和物流快递服务点135个，争创电子商务进农村全国示范市；抓好电商人才引进培养、快递物流体系建设和融资渠道拓展，用好阿里巴巴、京东等国内知名电商平台，鼓励龙头骨干企业自建电商平台，打造跨境电商示范基地，全市电子商务交易规模达到9亿元，增长20%。

　　（八）深化改革创新，激发发展活力。坚持激活内力与借助外力并重，抓住关键环节，破解发展难题，以改革增活力、以开放促开发、以创新促发展。

　　深入推进重点领域改革。持续深化“放管服”改革，取消和下放一批行政审批事项，加强事中事后监管，深化“双随机一公开”；加快推进网上审批系统全流程应用，推行“一网联通、一窗通办”，全面清理取消各种不合理证明和手续，公布审批事项证明清单，最大程度方便企业和群众。积极推动财税金融改革，推进财政事权和支出责任划分改革，深化预算制度改革，全面公开财政和部门“三公”经费预决算；创新投融资方式，发行债券10亿元，直接融资15亿元；大力发展普惠金融，引导金融机构服务实体经济，支持小微企业和“三农”发展；强化金融监管，深化“诚信平凉”建设，守住不发生区域性金融风险的底线。

　　全方位扩大开放开发。积极融入“一带一路”建设，推进全域开放与交流合作，主动对接中国（陕西）自由贸易试验区，培育对外贸易企业和产品品牌，进出口总额增长15%。启动红牛、苹果交易中心建设。加快发展会展经济，促进特色农产品、地域文化产品走进国内外大市场，实现区域、企业、产业融合发展。推进商事制度改革，建立市场准入负面清单制度，构建亲清新型政商关系，不断优化非公经济发展的政策、政务和法治环境，真心实意为非公企业排忧解难，激发非公经济发展活力，扩大民间投资比重。支持保险、担保等社会中介组织发展壮大，发挥“助保贷”等平台作用，着力解决中小微企业融资难融资贵问题，全市新增非公经济市场主体1.7万户。

　　大力实施创新驱动发展战略。加快科技创新体系建设，支持华煤、红峰、虹光等企业与科研院所建立产学研用长效合作机制，支持平凉中电科、超越农业等创建高新技术企业，提升核心竞争力；大力实施商标品牌战略，支持企业加快新产品、新技术开发，积极注册一批新商标，争创一批省级名牌产品和中国驰名商标，推进质量强市。加强创新人才培养，启动百名高端人才柔性引进工程，鼓励建立院士（专家）工作站（室），支持高校在我市建立技术转移中心，发挥甘肃医学院、平凉职业技术学院以及各县职教中心作用，推进脱贫攻坚人才保障和煤电化、红牛、金果等“1+8”人才培养工程，多渠道、多形式打造高科技人才、企业家、实用人才三支队伍。深入推进大众创业万众创新，建设中小企业创业园、青年创业基地等一批“园中园”，积极争创省级创业孵化基地，加快发展众创空间，支持科技人员、高校毕业生、返乡人士、乡土能人等重点群体因地制宜创业就业。

　　（九）保障改善民生，创造幸福美好生活。坚持以人民为中心的发展思想，抓住人民群众最关心最直接最现实的利益问题，一件事情接着一件事情办，不断提升广大群众的获得感、幸福感和安全感。

　　完善就业和社会保障体系。全面落实就业扶持政策，应届普通高校毕业生就业率达到85%以上。重视和加强农民工工作，维护农民工合法权益。推进全民参加社会保险计划，全面开展城乡居民异地就医直接结算。健全城镇职工养老保险和城乡居民基本养老保险制度，探索医养结合发展模式，完善居家、社区养老服务，放开养老服务市场，提升养老服务质量，市老年服务中心和市老年公寓建成投用，新建改扩建农村互助老人幸福院100个，为全市3万名80岁以上老年人办理意外伤害保险，为65岁以上老年人、农村建档立卡贫困人口开展免费健康体检。完善社会救助体系，落实城乡低保提标政策，做好特困供养对象、孤儿、高龄和失能老人、残疾人生活保障，关爱农村留守儿童、妇女和老人，支持慈善事业发展。

　　协调发展社会事业。围绕打造全国教育扶贫先行先试区，落实第三期学前教育行动计划，继续实施“全面改薄”工程，扶持办好农村小规模学校，新建改建校舍10.3万平方米，新建改扩建城镇幼儿园16所、新增园位5000个，全力解决城镇“入园难”“大班额”问题；崆峒区义务教育均衡发展通过国家评估认定；加快普及高中阶段教育，规划布局中心城区高中学校；抓好平凉职业技术学院三期、陕师大平凉实验中学续建。深化医药卫生体制改革，加快公立医院综合改革，完善医保体系，强化基本公共卫生服务，推行基层医疗卫生机构运行机制改革，完善以医联体为主要架构的市县乡村一体化服务体系，支持发展中医药事业，打造陕甘宁三省区区域性医养中心。建成市博物馆新馆，实施乡镇（社区）体育中心10个、村级农民健身工程120个，扶持民间协会和民营艺术团体发展，丰富群众性文体活动，提升全民健康素质。

　　创新加强社会治理。深化平安平凉建设，严格落实社会治安综合治理领导责任制，健全完善矛盾纠纷多元化解机制，畅通信访渠道，构建社会治安防控体系，依法打击和惩治黄赌毒黑拐骗等违法犯罪活动，打造共建共享共治的社会治理格局。加强安全生产监管信息化和应急救援能力建设，坚决遏制重特大事故发生。加强对环境安全、食品药品安全等重点领域薄弱环节的隐患排查和专项整治，提高公共安全水平。发展社会组织，推进“三社联动”。加强农村基层基础工作，健全自治、法治、德治相结合的乡村治理体系。加强社会主义核心价值观教育和社会公德、职业道德、家庭美德、个人品德建设，提升公民文明素养和社会文明程度。加大“天价彩礼”、薄养厚葬等问题整治力度，推动移风易俗。全面落实党的民族政策，深入推进民族团结进步及“两个共同”示范区创建活动。认真贯彻党的宗教工作基本方针，依法加强宗教事务管理，保持宗教和睦和顺。加强国防动员和双拥工作，支持工会、共青团、妇联、科协、残联等群团组织工作。加强地震、气象、外事、侨务、人防、老龄、地方志、档案、红十字会等工作。

　　建设人民满意的服务型政府

　　全面完成2018年各项目标任务，对政府工作提出了新的更高要求。我们将牢固树立“四个意识”，严格遵守政治纪律和政治规矩，按照“担当、创新、突破、提升”的工作要求，下功夫解决“知识恐慌” “本领恐慌”问题，认真履职尽责，对人民负责，受人民监督，让人民满意。

　　大力解放思想，提升改革创新能力。坚持用习近平新时代中国特色社会主义思想武装头脑，主动适应经济社会发展新形势、新变化，破除因循守旧、狭隘封闭思想，树立开拓创新意识，改变对原有发展路径、发展方式的依赖，推动思维创新、工作创新、机制创新；破除消极等待、小进即满思想，树立横向比较意识，跳出平凉看平凉，增强赶超进位的紧迫感和责任感；破除敷衍塞责、畏首畏尾思想，树立责任担当意识，不回避矛盾，不掩饰问题，不推卸责任，无私无畏促改革、抓发展。

　　强化责任担当，提升执行落实能力。进一步完善目标责任管理和绩效考核机制，健全正向激励机制，探索建立容错纠错机制，层层传导压力，常态化督促检查，确保中央和省市委决策部署落地生根。大力弘扬务实苦干精神，认真落实“俯身抬头”工作法，全面推行政府工作“三个清单”管理，坚决整肃懒政怠政不作为行为，把雷厉风行和久久为功有机结合起来，实干担当尽责，“钉钉子”、当“工匠”，切实提升抓落实的质量和效率。

　　规范施政行为，提升依法行政能力。把法治思维和法治方式贯穿于政府工作全过程，依法履行职责，主动接受人大法律监督、政协民主监督和社会舆论监督，认真做好人大、政协视察调研意见建议的落实和反馈，建立交办、领办、督办、现场办理机制，提高人大代表建议和政协委员提案办理质量。进一步完善政府议事规则及重大决策程序，健全决策咨询制度，落实法律顾问制度，提高政府决策的科学化民主化法治化水平。强化行政执法监督，推进严格规范公正文明执法。推动公共资源配置、重大项目实施、社会公益事业建设等领域政府信息全程公开、全程留痕，保障人民群众知情权、参与权和监督权。

　　加快职能转变，提升服务群众能力。深化行政管理体制改革，推动政府职能向创设良好环境、提供优质服务、维护社会公平正义转变。优化公共资源配置，加大财政对民生、“三农”、社会事业和公共设施的投入力度，努力为人民群众提供更多的优质公共产品。进一步简化办事程序，严格执行首问负责、限时办结、公开承诺、落实回告、责任追究制度，切实提高服务效能。认真解决群众关切的利益问题，真心实意为人民群众办实事、做好事、解难事，提高人民群众的满意度。

　　加强廉政建设，提升拒腐防变能力。严格落实全面从严治党主体责任，不断规范和加强政府系统党组工作。扎实推进“两学一做”学习教育常态化制度化，深入开展“不忘初心、牢记使命”主题教育，拓展丰富党建统领“一强三创”行动实效。严格落实中央八项规定精神和省市委有关规定，坚决纠正损害群众利益的不正之风，大力整治“四风”新表现新动向，驰而不息改进作风。健全权力运行制约和监督机制，加强国家监察和审计监督，严肃查处违纪违法案件，树立为民务实清廉的政府形象。

　　各位代表，立足新起点，展望新征程，我们深感责任重大，使命光荣。让我们紧密团结在以习近平同志为核心的党中央周围，在省委、省政府和市委的坚强领导下，不忘初心，牢记使命，一步一个脚印向前迈进，用实干铸就业绩，为建设绿色开放幸福美好新平凉作出新的贡献！
